# Knowledge, attitudes, and practices of pre-hospital emergency medical care practitioners regarding sepsis recognition: a cross-sectional study in Qatar

**DOI:** 10.1186/s12873-026-01607-7

**Published:** 2026-05-16

**Authors:** Michael Steven Paul Lewis, Paula J. W. Smith, Ian Lucas Howard, Hassan Farhat, Guillaume Alinier

**Affiliations:** 1 Hamad Medical Corporation Ambulance Service (HMCAS), Doha, Qatar; 2 The University of Edinburgh, Edinburgh, UK; 3 Edinburgh Medical School, The University of Edinburgh, Edinburgh, UK; 4 Department of Clinical Science and Education, Södersjukhuset, Karolinska Institutet, Solnavägen 1, Solna, 171 77 Sweden; 5 Division of Emergency Medicine, Stellenbosch University, Stellenbosch, South Africa; 6 Epidemiology of Mental Illnesses, Screening and Early Management Laboratory, Faculty of Medicine “Ibn El Jazzar”, University of Sousse, Sousse, 4054 Tunisia; 7 University of Hertfordshire, Hatfield, UK; 8 Weill Cornell Medicine – Qatar, Doha, Qatar; 9 Northumbria University, Newcastle Upon Tyne, UK

**Keywords:** Pre-hospital emergency care, Sepsis recognition, Knowledge, Attitudes, Practices, Emergency medical services, Qatar

## Abstract

**Background:**

Sepsis remains a leading cause of global morbidity. Early recognition is frequently delayed due to heterogeneous clinical presentations and the absence of definitive diagnostic tools, particularly in the pre-hospital setting. This study assessed the knowledge, attitudes, and practices (KAP) of pre-hospital emergency medical care (PHEMC) practitioners at Hamad Medical Corporation Ambulance Service (HMCAS) in Qatar to evaluate sepsis recognition and inform context-specific strategies for improving pre-hospital care.

**Methods:**

A cross-sectional study was conducted within HMCAS using a 53-item survey developed through literature synthesis, expert review, and pilot testing. Analyses were primarily descriptive, with exploratory bivariate testing of associations between practitioner characteristics and KAP measures. A multiple linear regression model was constructed to identify predictors of diagnostic confidence using confidence in prompt and accurate sepsis diagnosis as the dependent variable.

**Results:**

Of the 221 licensed practitioners who completed the survey, most were male (90%) and aged 31–40 years. Only 33.5% of respondents correctly determined that the patient in Scenario 1 did not have sepsis, whereas 79.2% correctly identified sepsis in Scenario 2, which represented a more overt septic presentation. Although 59.7% reported considering sepsis even in the absence of fever or a clear source of infection, scenario-based accuracy did not reflect this self-reported awareness. Awareness of screening tools was common, but their use was predominantly reactive, occurring after clinical suspicion had already been established. Diagnostic confidence was highest among practitioners who had received recent/up-to-date sepsis training (*p* < 0.001).

**Conclusion:**

Despite widespread awareness and training, early-stage sepsis recognition among PHEMC practitioners in Qatar remains inconsistent and largely reactive. Self-reported confidence and familiarity with screening tools did not reliably translate into accurate determination of whether sepsis was present, highlighting a gap between perceived and actual competence. These findings suggest that improving pre-hospital sepsis recognition may require system-level and behavioural interventions in addition to education.

**Supplementary Information:**

The online version contains supplementary material available at 10.1186/s12873-026-01607-7.

## Introduction

Sepsis, a life-threatening dysregulated response to infection, triggers a complex and often deadly race between invading pathogens and immune response dysregulation [1]. Despite improvements in treatment, sepsis remains a global health crisis [2] and a formidable challenge in emergency medical care [3]. International evidence indicates that in-hospital mortality for sepsis ranges from 20 to 30%, and for septic shock from 30 to 40%, depending on severity and health system context [4, 5]. These rates exceed those associated with other time-sensitive emergencies, such as myocardial infarction and stroke [6, 7]. Timely sepsis recognition and treatment are therefore central to reducing sepsis-related deaths, as survival gains in recent years have been attributed primarily to earlier recognition and prompt initiation of care, rather than the development of novel therapeutic agents [8]. While demographic variables, including age and sex, influence sepsis presentation and outcomes [9], diagnosis remains challenging due to clinical and biological heterogeneity, overlap with other acute conditions [10–14], and the absence of definitive diagnostic tests [15]. This nonspecific presentation complicates diagnosis, particularly when compared to more linear diagnostic pathways seen in conditions like ST-elevation myocardial infarction (STEMI) or stroke [16], necessitating a proactive recognition strategy in the pre-hospital environment [17].

Pre-hospital emergency medical care (PHEMC) practitioners are the first point of contact for approximately 50–75% of severely ill sepsis patients, positioning them critically for early recognition and treatment, factors linked to reduced mortality [7, 18]. Contemporary Emergency Medical Services (EMS) cohort data from North America further illustrate this burden: sepsis accounts for approximately 2.1% of EMS transports, with affected patients experiencing an in-hospital mortality rate of 28% [19]. A 2021 German cohort study demonstrated that pre-hospital identification of sepsis was associated with a more than twofold reduction in 30-day mortality (8.0% vs 22.8%) [20]. However, sepsis in the pre-hospital setting remains comparatively under-researched relative to other time-critical conditions.

Key challenges in the pre-hospital environment include nonspecific symptoms, the absence of definitive diagnostic tools and the lack of a universally validated pre-hospital screening instrument [21, 22]. Although several screening instruments—such as qSOFA, PRESS, PRESEP, and MEWS—have been proposed to support early sepsis recognition, their diagnostic performance has been highly inconsistent [6, 21]. A recent scoping review encompassing 23 studies reported sensitivities ranging from 0.02 to 1.00 and specificities from 0.07 to 1.00, concluding that a single “gold-standard” score is unlikely to emerge as a universally applicable solution. Large UK ambulance cohort work from the Pre-Hospital Early Warning Score (PHEWS) programme reinforced this limitation, demonstrating that no combination of early warning score and paramedic diagnostic impression could simultaneously deliver high sensitivity and acceptable positive predictive value, revealing an inherent trade-off between missed cases and over-triage [23]. Consequently, early warning systems should be considered adjuncts to clinical judgement rather than standalone tools [24]. Observational EMS studies further show that pre-hospital suspicion or documentation of sepsis remains low (6–36%) among patients later diagnosed in hospital [18, 19, 25, 26]. Collectively, this evidence indicates that neither existing screening instruments nor current recognition practices are sufficient on their own, shifting emphasis toward the human factors that shape sepsis recognition, particularly PHEMC practitioners’ knowledge, attitudes, and practices.

Within this context, several cross-sectional studies have examined the KAP of PHEMC practitioners regarding sepsis management [8, 27–31]. These studies have largely focused on general sepsis awareness and definitions, recognition of key signs and symptoms, and familiarity with management principles, rather than on early recognition as a primary outcome. Most have identified gaps in knowledge and uncertainty about appropriate pre-hospital management strategies, particularly among less experienced or less highly qualified practitioners, although some cohorts have demonstrated satisfactory knowledge in selected domains [28, 31]. Beyond knowledge deficits, attitudinal and behavioural barriers have been described, including limited appreciation of the pre-hospital contribution to early triage and variable engagement in sepsis care [8, 27, 31]. To address these gaps, researchers have proposed targeted sepsis education, refinement of diagnostic protocols, and system-level toolkits to support early recognition and pre-notification [8, 27, 30, 31]. However, existing literature has yet to conclusively determine which aspects of knowledge, attitude, or practice most strongly influence pre-hospital sepsis recognition in routine clinical environments, nor how these factors operate within highly developed EMS systems. This limits services’ ability to design focused training, protocol development, and system interventions that align with the realities of pre-hospital decision-making and the complexity of early sepsis recognition [22, 23, 32].

Qatar, with its diverse population, presents a unique and valuable context in which to study PHEMC practitioners’ KAP related to sepsis. As a rapidly advancing healthcare system with robust emergency services, Qatar has invested significantly in pre-hospital infrastructure, led by the Hamad Medical Corporation Ambulance Service (HMCAS) [21]. HMCAS is a mature, nationally coordinated PHEMC system staffed by highly qualified practitioners with access to advanced pre-hospital diagnostic and monitoring tools. Despite these developments, no published studies have examined pre-hospital sepsis recognition within Qatar’s EMS system. Investigating sepsis-related KAP in this context, therefore, provides an opportunity to extend international work and build on the only regional EMS study from Saudi Arabia, which assessed general sepsis knowledge and attitudes but did not evaluate early recognition or applied decision-making. By focusing on applied early recognition within a high-capacity, regulated EMS system, this study addresses a critical gap in regional and global literature. This emphasis on human and system-level factors is increasingly important, as recent integrative reviews highlight the very limited use of EMS clinical data to support early emergency department sepsis identification, including the absence of models that can enhance pre-hospital detection through machine learning approaches [33].

This study, therefore, aimed to describe the KAP of HMCAS practitioners regarding sepsis recognition and to identify practitioner- and training-related factors associated with self-reported diagnostic confidence. In addition, the study explores applied recognition using scenario-based assessments, providing insight into how practitioners determine whether sepsis is present in clinically contrasting pre-hospital presentations. The findings are intended to inform targeted, context-specific strategies to strengthen pre-hospital sepsis recognition in Qatar and comparable EMS systems.

## Methods

### Study design

This descriptive, cross-sectional study was conducted in Qatar to explore HMCAS practitioners’ KAP regarding sepsis recognition. An anonymous online questionnaire was administered between 10 October and 25 November 2023. This design was selected to estimate the distribution of knowledge, attitudes, and practices, as well as applied sepsis recognition performance, within a defined group of healthcare professionals, at a single point in time [34]. The study is reported in line with Strengthening the Reporting of Observational Studies in Epidemiology (STROBE) guidelines [35].

### Setting and recruitment of the study sample

The study was conducted within HMCAS, Qatar’s sole national provider of PHEMC. HMCAS delivers 999 emergency responses and inter-facility transports across all regions of Qatar via a hub-and-spoke deployment model, thereby representing the national PHEMC system. It employs three clinician categories with different levels of clinical practice: ambulance paramedics (APs), critical care assistants (CCAs), and critical care paramedics (CCPs) [36]. While APs and CCAs share similar clinical levels, CCPs operate at a more advanced clinical level. For analysis, participants were grouped into CCA/AP and CCP cohorts [37].

A total-population strategy was used, inviting all HMCAS PHEMC practitioners employed in Qatar as APs, CCAs, and CCPs (*N* = 1,021; 110 CCP, 911 AP/CCA). Invitation emails were sent via the corporate email system to HMCAS staff lists restricted to these clinical cadres. This included practitioners temporarily assigned to training or administrative roles, provided they maintained licensure as PHEMC practitioners within HMCAS. Other sectors of the Qatari healthcare system (e.g. hospital-based and primary care services) were not included because the research question focused specifically on pre-hospital sepsis recognition among PHEMC practitioners, all of whom are employed within HMCAS.

A precision-based sample size estimation for a finite population was undertaken [38], assuming maximum variability (*p* = 0.5), a 95% confidence level (α = 0.05), and a 5% margin of error, yielding a minimum required sample of *n* = 288. This approach is appropriate for descriptive surveys that estimate population proportions with a defined precision rather than testing prespecified hypotheses. A formal power calculation was not undertaken, as the primary objective was descriptive estimation rather than hypothesis testing.

Inclusion criteria were [1]: employment as an AP, CCA, or CCP within HMCAS in Qatar at the time of the survey; and [2] receipt of the electronic survey invitation via an HMCAS email account. Exclusion criteria were [1]: failure to provide informed consent; and [2] questionnaire completion of <80% of items. Recruitment was conducted via corporate personnel email, SMS and QR-coded posters at ambulance stations. To ensure data security, all responses were submitted via a secure online platform. The online survey remained open for six weeks (10 October to 25 November 2023) with a weekly reminder email sent to the target population.

Of the 1,021 clinicians contacted, 224 responded (21.9%), and 221 were included in the final analysis (21.6%). The achieved sample was below the estimated minimum and may be associated with reduced precision and potential non-response bias, as responder characteristics may differ from non-responders. Due to survey anonymity, non-responder data were unavailable.

### Instrument development and content

A 53-item self-administered questionnaire was developed through a three-stage process. First, a literature synthesis was undertaken on sepsis-related KAP among EMS providers [8, 27–30, 39] and on contemporary sepsis guidelines (e.g., Surviving Sepsis Campaign) [15]. Second, a structured expert review was conducted with seven academic and clinical specialists in emergency care and sepsis, who evaluated item clarity, relevance, and content coverage. Third, the draft instrument was piloted with frontline HMCAS practitioners to assess face validity, acceptability, and completion time; minor wording modifications were made in response to feedback.

The final instrument (see Appendix A) was completed anonymously and covered four domains: demographics (8 items), knowledge (18 items), attitudes (12 Likert-scale items), and practices (15 items). It included two scenario-based vignettes to assess applied knowledge in sepsis recognition. Items were presented in multiple-choice, dichotomous, and ranked formats. Response options for all items are provided in the supplementary instrument. Scoring and derivation of domain-level summary measures are described separately in the Statistical Analysis and KAP Scoring sections.

### Validity and reliability

Content validity was assessed through an expert review involving seven academic and clinical specialists in emergency care and sepsis who were not part of the study sample. Experts independently rated survey items for clarity and relevance. Aiken’s V was then calculated to quantify the level of agreement on item clarity and relevance, and domain-level Content Validity Indices (CVI) were computed for each construct [40].

The resulting indices indicated acceptable overall content validity across domains, all with domain-level CVI values meeting or exceeding the commonly recommended minimum threshold of 0.78 for panels of six to ten experts [41, 42], including the Attitude domain (relevance CVI = 0.79) (Table 1).

**Table 1:** Scale-level content validity index (CVI)

Constructs	Clarity	Suitability (Yes/No)	Relevance	Suitability (Yes/No)
Demographics CVI	0.84	Yes	0.80	Yes
Attitude CVI	0.85	Yes	0.79	Yes
Practice CVI	0.90	Yes	0.91	Yes
Knowledge CVI	0.89	Yes	0.91	Yes
Scale Level CVI	0.87	Yes	0.85	Yes

Footnote: The accepted minimum I-CVI threshold for panels of six to ten experts is ≥0.78; therefore, the Attitude relevance CVI of 0.79 met this threshold despite narrowly falling below the stricter 0.80 criterion sometimes applied in the literature [42]

Internal consistency reliability of the attitude and practice scales was evaluated using Cronbach’s alpha based on participant responses from the study sample, to assess the coherence of items within each domain [38]. These findings should be interpreted as preliminary measures of internal consistency within this single-sample context.

### Data collection and management

Data were collected anonymously via the Online Survey platform. Participants provided informed consent digitally before accessing the questionnaire. Responses were exported into IBM SPSS Statistics v27 (IBM Corp., Armonk, NY, USA) for cleaning and analysis.

### Statistical analysis

Descriptive statistics summarised participants’ characteristics and responses across the KAP domains (Table 2). Knowledge scores were classified as poor (<50%), adequate (50–75%), or excellent (>75%) (Table 3), consistent with the scoring framework applied in previous KAP studies [43, 44]. Attitude and practice items measured on 5-point Likert scales (1 = *strongly disagree* to 5 = *strongly agree*) were reported descriptively as frequencies and percentages for each response option. Chi-square tests were used to compare categorical variables.

**Table 2:** Demographic data of the study participants

	Question	Overall	
		n	%
1	Age category (Years)		
	20–30	20	9.0
	31–40	155	70.1
	41–50	35	15.8
	Over 50	11	5.0
2	Sex		
	Male	199	90.0
	Female	20	9.0
	Choose not to disclose	2	0.9
3	Are you currently practising as a pre-hospital emergency medical care (PHEMC) practitioner in Qatar?		
	Yes	217	98.2
	No	4	1.8
3a	How would you qualify your primary role/function at Hamad Medical Corporation Ambulance Service (HMCAS)?		
	Administrative/Support	10	4.52
	Operations	172	77.83
	Training	10	4.52
	Other	29	13.12
4	Are you registered with the medical board in Qatar (Qatar Council for Healthcare Practitioners (QCHP))?		
	Yes	218	98.6
	No	3	1.4
5	What is your current scope/level of practice?		
	Ambulance Paramedic (AP)/Critical Care Assistant (CCA)	176	79.6
	Critical Care Paramedic (CCP)	45	20.4
6	How many years of experience in total do you have as a licensed PHEMC practitioner?		
	2 or less years	15	6.8
	3–5 years	51	23.1
	6–10 years	76	34.4
	11 or more years	79	35.7
6a	How many of these years of experience as a licensed PHEMC practitioner were gained while working in Qatar?		
	2 or less years	36	16.3
	3–5 years	72	32.6
	6–10 years	88	39.8
	11 or more years	25	11.3
7	What is the highest medical qualification you hold?		
	No formal qualification	1	0.5
	Diploma	18	8.1
	Associate degree	3	1.4
	Bachelor’s degree	162	73.3
	Master’s degree	31	14.0
	Doctor of Philosophy (PhD)	2	0.9
	Other	4	1.8
8	In addition to your current licensure as a PHEMC practitioner, have you ever been or are currently licensed to practice in any other medical profession?		
	Yes	72	32.6
	No	149	67.4
8a	Do you have any speciality in which you have practised within this other profession?		
	Yes	29	40.3
	No	43	59.7
	N-Miss	149	
9	Within your training or education as a healthcare professional, have you received training to recognise patients with sepsis?		
	Yes	167	75.6
	No	28	12.7
	Unsure	26	11.8
9a	What is the level of training or education you have received on sepsis recognition?		
	Basic (1/2-day course)	84	50.3
	Intermediate (2-day course)	30	18.0
	In-depth (full university module)	36	21.6
	N-Miss	54	
	Unsure	17	10.2
9b	When did you last receive training on sepsis recognition?		
	Within the last 6 months	45	26.9
	Within the last year	51	30.5
	Within the last 2 years	27	16.2
	More than 2 years ago	25	15.0
	N-Miss	54	
	Unsure	19	11.4
10	Other healthcare profession		
	Nurse	55	76.4
	Technician	6	8.3
	N-Miss	149	
	Other	11	15.3

*Note. N* = 221, *N*-Miss indicates missing or non-applicable responses due to branching logic

Abbreviations: PHEMC: Pre-hospital emergency medical care; HMCAS: Hamad Medical Corporation Ambulance Service; QCHP: Qatar Council for Healthcare Practitioners; AP: Ambulance paramedic; CCA: Critical care assistant; CCP: Critical care paramedic; PhD: Doctor of Philosophy

^*^A statistically significant association was found between clinical role and sepsis training, with CCPs more likely to have received training (χ^2^ [2] = 15.205, *p* < 0.001)

^**^Total years of experience were significantly associated with experience gained in Qatar (χ^2^ [9] = 188.724, *p* < 0.001)

**Table 3:** Knowledge assessment of study participants on sepsis recognition and management

Reference	Question	Total Respondents	Correct Responses	% Correct	Knowledge Level
Q21	Do you think the patient presented to you in Scenario 1 has sepsis?	221	74	33.5	Poor
Q22a	Do you feel it is necessary to call CCP for backup for this patient?	173	104	60.12	Adequate
Q22b	Do you think it is necessary to administer Noradrenaline to this patient?	48	28	58.33	Adequate
Q23	Do you think the patient presented to you in Scenario 2 has sepsis?	221	175	79.19	Excellent
Q26	Would you still consider the possibility of sepsis in any of these two patients if the patients did not present with fever and an obvious source/history of infection?	221	132	59.73	Adequate
Q29	Considering the scenarios of the two patients you treated, do you think obtaining blood cultures and serum lactate levels in the pre-hospital setting would be beneficial in these patients’ chain of care?	221	116	52.49	Adequate
Q30	If a patient presents with sepsis, it is common practice today to categorise them into the following different levels, namely “sepsis”, “severe sepsis”, and “septic shock”.	221	26	11.76	Poor
Q31	Do you believe that if a patient with sepsis is not treated, they will almost certainly develop septic shock?	221	12	5.4	Poor
Q32	Generally, which treatments are essential for managing patients with sepsis?	221	162	73.3	Adequate
Q33	I believe sepsis has a higher global mortality rate than the following conditions: Lung Cancer, Stroke, Major Trauma, Myocardial Infarction, COPD (Chronic obstructive pulmonary disease)	221	141	63.8	Adequate
Q34	When do you think sepsis in patients is often first suspected/identified?	221	88	39.82	Poor
Q36	Do you agree with the statement: The management of sepsis patients is time-critical, irrespective of their specific condition?	221	197	89.14	Excellent

*Note*. Descriptive comparisons showed some variation in knowledge scores across education levels, years of experience, and scope of practice. However, no statistically significant associations were identified between overall knowledge and these variables. Q22a and Q22b were conditional items based on participants’ responses to the preceding scenario question; therefore, denominators differ from the full sample.

Differences in KAP scores across demographic and professional subgroups were assessed using Mann–Whitney U tests (two-group comparisons) and Kruskal–Wallis H tests (three or more groups), with pairwise Mann–Whitney U tests conducted for post hoc comparisons where overall tests were statistically significant (Table 4).

**Table 4:** Statistically significant differences in knowledge, attitude, and practice outcomes by demographic and professional groupings (non-parametric tests)

Domain	Variable	Test	Significant Differences (p < 0.05)	Questions with Significant Differences
Knowledge	Scope of Practice (AP/CCA vs CCP)	Mann–Whitney U	Yes	Q32 (*p* = 0.003), Q34 (*p* < 0.001)
Knowledge	Highest qualification level	Kruskal–Wallis H	No	–
Knowledge	Total years of PHEMC experience	Kruskal–Wallis H	Yes	Q23 (*p* = 0.007), Q34 (*p* = 0.035)
Knowledge	Years of experience in Qatar	Kruskal–Wallis H	Yes	Q30 (*p* = 0.008)
Knowledge	Sepsis training level	Kruskal–Wallis H	No	–
Attitude	Scope of practice (AP/CCA vs CCP)	Mann–Whitney U	Yes	Q2.1.1 (*p* < 0.001), Q2.1.2 (*p* < 0.001), Q2.1.3 (*p* < 0.001), Q2.1.4 (*p* < 0.001), Q2.1.7 (*p* < 0.001), Q2.1.9 (*p* < 0.001), Q2.1.10 (*p* = 0.006), Q2.1.11 (*p* = 0.031), Q2.1.12 (*p* = 0.020)
Attitude	Total years of experience	Kruskal–Wallis H	Yes	Q2.1.11 (*p* = 0.028), Q2.1.12 (*p* = 0.040)
Attitude	Highest qualification level	Kruskal–Wallis H	No	–
Attitude	Training received on sepsis	Kruskal–Wallis H	Yes	Q2.1.1 (*p* = 0.013), Q2.1.2 (*p* = 0.025)
Practice	Scope of practice (AP/CCA vs CCP)	Mann–Whitney U	Yes	Q12a (*p* < 0.001), Q16 (*p* = 0.008)
Practice	Total years of experience	Kruskal–Wallis H	No	–
Practice	Highest qualification level	Kruskal–Wallis H	Yes	Q13 (*p* = 0.019)
Practice	Training received on sepsis	Kruskal–Wallis H	Yes	Q14 (*p* = 0.007), Q16 (*p* = 0.010)

This study aimed to describe the knowledge, attitudes, and practices of HMCAS practitioners regarding sepsis recognition and to explore factors influencing scenario-based determination of whether sepsis was present in contrasting pre-hospital presentations. To improve analytic clarity, the primary endpoint was defined as accuracy of sepsis recognition across the two scenario-based vignettes, based on whether respondents correctly determined if sepsis was present or absent. Scenario 1 assessed respondents’ ability to recognise that the patient did not have sepsis, whereas Scenario 2 assessed recognition of sepsis in a more overt presentation Domain-level KAP scores and individual item responses were analysed as secondary or exploratory outcomes. Given the number of item-level and subgroup comparisons performed, these analyses were interpreted cautiously without formal adjustment for multiple testing.

To examine factors associated with diagnostic confidence, an exploratory multiple linear regression model was fitted. Diagnostic confidence was operationalised using Q1 (“As a PHEMC practitioner, you are confident in your ability to make a sepsis diagnosis promptly and accurately”) as the dependent variable. Because this item was measured on a 5-point Likert scale and analysed as an approximately continuous outcome, linear regression was used for exploratory modelling. Predictor variables were selected a priori from attitude items theoretically relevant to diagnostic confidence (Table 5).

**Table 5:** Regression analysis of predictors of diagnostic confidence in sepsis recognition (dependent variable: Q1)

Predictor Variable	Unstandardized B	SE	Standardized β	t	*p*-value
Recent/up-to-date sepsis training	0.385	0.047	0.416	8.12	<0.001
Belief in the importance of PHEMC in sepsis care	0.295	0.057	0.320	5.16	<0.001
Belief in the value of pre-notification	0.214	0.048	0.240	4.45	<0.001
Previous potential missed diagnosis	−0.053	0.035	−0.066	−1.54	0.126

*Note*. Model R^2^ = 0.657, F (4,216) = 103.60, *p* < 0.001**. Dependent variable: Q1 (“As a PHEMC practitioner, you are confident in your ability to make a sepsis diagnosis promptly and accurately”). Predictors entered simultaneously using the enter method were Q2 (recent/up-to-date sepsis training), Q3 (belief in the importance of PHEMC in sepsis care), Q8 (belief in the value of pre-notification), and Q6 (previous potential missed diagnosis). Collinearity was low (VIF 1.171–2.425), and residual independence was acceptable (Durbin–Watson = 1.916).**

Predictor variables were selected a priori based on their theoretical and empirical relevance to the KAP model, consistent with established KAP research methodology and recent best-practice guidance [45, 46]. Specifically, the model included [1]: Recent training on sepsis recognition (Q2) [2]; Belief in PHEMC’s role in patient outcomes (Q3) [3]; Perceived benefit of pre-notification (Q8) [4]; Experience of previously missed sepsis case (Q6). All four predictors were entered into the model simultaneously using the enter method; no stepwise or other data-driven selection procedures were used. Categorical predictors were coded so that higher values reflected greater exposure or stronger agreement with the underlying statement. Model diagnostics were examined to assess the suitability of linear regression. Residual independence was acceptable (Durbin–Watson = 1.916), and collinearity was low (tolerance 0.412–0.854; VIF 1.171–2.425). Standardised residuals, histograms, normal probability plots, and ZPRED-versus-ZRESID plots were inspected to assess residual normality and homoscedasticity, and no violations were observed.

All analyses were conducted using IBM SPSS Statistics v27 (IBM Corp., Armonk, NY, USA), with statistical significance set at *p* < 0.05 (Table 5).

### KAP scoring and summary statistics

For summary reporting, mean domain scores were calculated as follows: the knowledge score is the proportion of correct responses across all knowledge items; the attitude and practice scores are the mean of responses to all items presented on a 5-point Likert scale. Only Likert-scale items were included in the calculation of mean attitude and practices scores, as non-Likert or categorical items were not suitable for averaging.

In addition to these summary measures, all KAP items, regardless of response format, were analysed and reported individually to preserve interpretive completeness and ensure methodological consistency.

### Ethics

Ethical approval for this study was obtained from the University of Edinburgh Medical School Research Ethics Committee (ML291122), the Hamad Medical Corporation Institutional Review Board (MRC-01–23-406), and the Academic and Clinical Central Office for Research and Development (ACCORD), University of Edinburgh (Sponsor Reference: AC23033).

## Results

Of the 224 survey responses received, three were excluded due to incomplete submission (<80% completion), resulting in 221 valid responses included in the analysis (Table 2). The instrument demonstrated acceptable content validity (Aiken’s V: 0.81 to 0.89; scale-level clarity 0.87; relevance 0.85) (Table 1) , and the attitude and practice scales demonstrated good internal consistency based on Cronbach’s α= 0.89. 

Participants were predominantly male and mid-career, with most actively practising in frontline PHEMC roles at the time of the survey. Regarding clinical designation, 79.6% were either APs or CCAs, while 20.4% were CCPs. For analysis, APs and CCAs were grouped due to overlapping frontline roles. More than one-third (35.7%) of respondents reported over 11 years of clinical experience, and 39.8% had been working in Qatar for between 6 and 10 years (Table 2).

### Knowledge of sepsis

Sepsis recognition training was reported by 75.6% of respondents.

The overall mean knowledge score among PHEMC practitioners was 57.5% (*SD* 14.7%; range 8–92%), situating the cohort within the study’s predefined *adequate* range (50–75%). However, performance was highly variable across individual items. While the majority (89.1%) recognised sepsis as a time-critical emergency and selected appropriate treatment principles (73.3%), there were marked deficits in applied knowledge domains. For example, only 33.5% of respondents correctly determined in the first scenario-based item (Q21) that the patient did not have sepsis, while just 11.8% selected the correct contemporary classification, and only 5.4% accurately identified the disease’s potential progression (Q31).

**Fig. 1:**
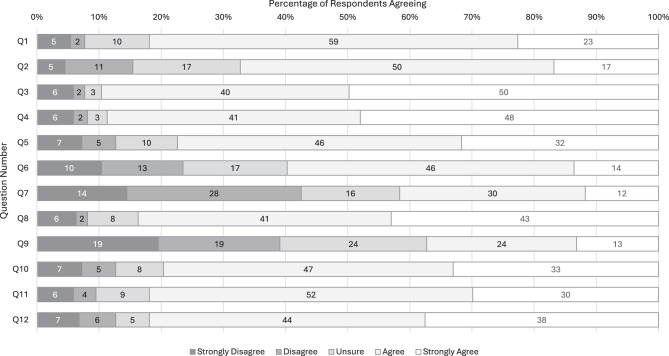
Attitudes of study participants towards sepsis recognition. Note: Question number: Q1: As a PHEMC practitioner, you are confident in your ability to make a sepsis diagnosis promptly and accurately. Q2: you have received up-to-date training on sepsis recognition, consistent with recognised standards or guidelines, which makes you confident in your recognition and management of patients with sepsis. Q3: within the chain of care for sepsis patients, pre-hospital emergency medicine is a vital link impacting patient outcomes. Q4: the accurate diagnosis and management of sepsis patients by you, as a PHEMC practitioner in the pre-hospital setting, significantly impacts sepsis patient outcomes. Q5: you believe that communicating your suspicion of sepsis to the emergency department (ED) staff will be taken seriously and can positively influence the quality and timeliness of patient care by the ED staff. Q6: because it is not a diagnosis commonly thought of, it is possible that you could have missed a patient having sepsis, even in the presence of clear signs and symptoms. Q7: you sometimes feel reluctant to make a diagnosis of sepsis or communicate your suspicion of sepsis in a patient due to the fear of potentially being wrong and, therefore, facing judgment. Q8: pre-notification of a sepsis patient to the receiving hospital can improve patient care and outcome. Q9: the administration of antibiotics for the treatment of sepsis should be implemented in pre-hospital emergency medicine. Q10: As a healthcare practitioner, when you suspect sepsis in a patient, it is necessary for you to have the same sense of urgency and considerations for diagnosis, treatment, and transportation as you would have for a patient with an ST-elevation myocardial infarction (STEMI) or stroke. Q11: As a PHEMC practitioner, you feel you require more knowledge and training regarding sepsis recognition. Q12: you consider sepsis (not specifically septic shock) a life-threatening medical emergency

For the primary outcome, 33.5% of respondents correctly determined that the patient in Scenario 1 did not have sepsis, whereas 79.2% correctly identified sepsis in Scenario 2, which represented a more overt septic presentation. Most respondents reported relying primarily on clinical presentation (93.7%), clinical judgement (72.4%), and personal experience (70.6%) to guide decision-making, rather than structured criteria (See Appendix B, Table 1). Although 43% reported that a key diagnostic indicator for sepsis is a specific sepsis criterion, such as qSOFA (See Appendix B, Figure 1), just over half of the participants reported routinely applying any formal screening tools in clinical practice (Table 6). Awareness of alternative screening tools was limited (see Appendix B, Figure 2).

**Table 6:** Participants’ response to survey questions related to their reported use and application of sepsis screening tools, to their pre-notification policy awareness and practice, and to the factors perceived as influencing sepsis recognition and suspicion

Question	Response	Frequency	%
Are you currently using any screening tool to aid your diagnosis of sepsis in the pre-hospital clinical setting?	Yes	125	56.6
	No	96	43.4
Do you apply the screening tool to every patient or only to patients you suspect might have sepsis? (Based on participants making use of screening tools *N* = 125)	All patients	26	20.8
	Suspected sepsis patients only	99	79.2
In patients who are critically ill with an undifferentiated diagnosis, sepsis is one of your top three differential diagnoses.	Strongly disagree	9	4.1
	Disagree	10	4.5
	Unsure	58	26.2
	Agree	123	55.7
	Strongly agree	21	9.5
A clear guideline indicating which patients should be screened for sepsis should be made available to PHEMC practitioners.	Yes	184	83.3
	No	10	4.5
	Makes no difference	13	5.9
	Unsure	14	6.3
Does the current system you work in have a formal policy or procedure for pre-notifying the receiving hospital about a suspected sepsis patient?	Yes	98	44.3
	No	73	33.0
	I don’t know; I’m willing to learn	50	22.6
As a PHEMC practitioner, do you pre-notify the receiving healthcare facility of a patient with suspected sepsis?	Never	32	14.5
	Rarely	45	20.4
	Sometimes	57	25.8
	Often	34	15.4
	Always	53	24.0
Diagnosing sepsis is difficult as the clinical signs and symptoms are often nonspecific.	Strongly disagree	4	1.8
	Disagree	47	21.3
	Unsure	31	14.0
	Agree	122	55.2
	Strongly agree	17	7.7
What factors do you think primarily heighten your index of suspicion for sepsis? (Select all that apply)	Patient’s clinical presentation	207	93.7
	Experience	156	70.6
	Clinical judgement	160	72.4
	Sepsis screening tool	149	67.4
	Source of infection	153	69.2
	Patient’s response to treatment	98	44.3
	Other	11	5.0
From your experience, what real factors may influence sepsis recognition in the pre-hospital clinical setting by PHEMC practitioners? (Select all that apply)	Training and Education	206	93.2
	Practitioner Attitude	96	43.4
	Practitioner experience	173	78.3
	Contact time between the patient and practitioner	87	39.4
	Practitioner awareness	158	71.5
	Availability of diagnostic tools	150	67.9
	Complexity of sepsis	95	43.0
	None	3	1.4
	Other	5	2.3
Would you say you may tend to diagnose septic shock more frequently than cases of only sepsis, as the symptoms of septic shock are typically more evident and recognisable?	Yes	159	71.9
	No	28	12.7
	Unsure	34	15.4

*Note*. The survey responses of Qatar’s PHEMC practitioners (*N* = 221)

Abbreviations

PHEMC: Pre-hospital Emergency Medical Care

Inferential analyses using the Mann-Whitney U and Kruskal-Wallis tests were conducted to further explore differences in knowledge, attitude, and practice domains across demographic and professional variables (Table 4). Statistically significant differences were observed in selected knowledge items across clinical groups and experience levels (Table 4). However, no statistically significant differences were observed for overall knowledge scores or by sepsis training level or highest qualification.

### Attitudes

Most participants (81.9%) reported confidence in their ability to recognise sepsis in the pre-hospital setting. However, the same proportion also expressed a need for further training, and only 66.0% felt their current education aligned with international standards (Fig. 1)

Inferential analysis identified multiple significant differences in attitude items by scope of practice (AP/CCA vs CCP), total years of experience, and sepsis training received. Practitioners with higher clinical scope, greater experience, and recent training demonstrated higher agreement with key statements on early recognition. However, these significant differences were not uniform across all attitude items, and overall attitude scores were broadly similar between groups.

- Regression Analysis (Diagnostic Confidence)

A multiple linear regression was conducted to identify factors associated with diagnostic confidence, using Q1 as the dependent variable. The model explained 65.7% of the variance in self-reported diagnostic confidence (*R*^*2*^ = 0.657, *F* (4,216) = 103.60, *p* < 0.001). Residual independence was acceptable (Durbin–Watson = 1.916), and collinearity was low (VIF 1.171–2.425).

Significant predictors included recent, up-to-date sepsis recognition training (Q2) (B = 0.385, *p* < 0.001), recognising the vital role of pre-hospital emergency care in sepsis management (Q3) (B = 0.295, *p* < 0.001), and believing that pre-notification improves patient outcomes (Q8) (B = 0.214, *p* < 0.001). Having previously missed a suspected sepsis case (Q6) showed a non-significant negative association with diagnostic confidence (B = −0.053, *p* = 0.126).

Most respondents recognised the urgency of early sepsis care: 83.7% agreed that pre-notification improves outcomes, and 79.6% believed sepsis warrants the same priority as STEMI or stroke. The majority (81.9%) viewed sepsis, beyond just septic shock, as a life-threatening emergency.

### Practices

The overall mean practice score, based on Likert-scale items, was 3.41 (*SD* = 0.68) out of 5, reflecting moderate engagement with recommended sepsis recognition behaviours among participants. A total of 56.6% of participants reported using sepsis screening tools in clinical practice (Table 6).

Among these, 79.2% applied the tools selectively (only when sepsis was already suspected), indicating that screening was typically confirmatory rather than proactive. qSOFA was the most frequently reported tool (30.9%), while awareness and use of other screening instruments, such as PRESS, PRESEP, and PreSAT, were low.

Significant differences in reported practice behaviours were observed by scope of practice (AP/CCA vs CCP), highest qualification level, and sepsis training received. Specifically, CCPs were more likely to apply screening tools selectively and to pre-notify receiving hospitals (Q12a, *p* < 0.001; Q16, *p* = 0.008). Higher qualifications and training were associated with greater engagement in key sepsis recognition practices.

While 65.2% of respondents reported including sepsis among their top three differential diagnoses in undifferentiated critical illness, only 44.3% stated that their service had a formal pre-notification policy. Participants aware of such policies were significantly more likely to always pre-notify the receiving hospital (39.8%) compared to those unaware (6.8%) χ^2^ = 54.62, *df* = 4, *p* < 0.001. Participants reported more frequent identification of septic shock than sepsis alone. Training, experience, and awareness were commonly identified as influencing sepsis recognition (Table 6).

## Discussion

The study population comprised a mature, professionally diverse cohort of PHEMC practitioners, most with considerable clinical experience within Qatar’s regulated EMS system (Table 2) [47]. Educational attainment was notably high, with most participants holding at least a bachelor’s degree, and a significant subset reporting postgraduate qualifications. Additionally, nearly one-third of the cohort were dually licensed in other healthcare professions, mostly nursing, with complementary training provided by HMCAS to align their qualifications with the APs/CCA clinical level. This multidisciplinary background, combined with the experience and scope of their professional roles, distinguishes this cohort from those in similar international studies, where EMS providers often had less formal education [28, 29], shorter service durations [8], and more narrowly defined scopes of practice [8, 27, 31]. These features provide an essential lens for interpreting the study’s findings and underscore the importance of considering the local workforce context when comparing to other EMS settings.

While prior sepsis training and awareness of key concepts were widespread, applied scenario-based determination of whether sepsis was present remained inconsistent. Respondents were substantially better at identifying sepsis in the more overt scenario than correctly determining that sepsis was absent in the first scenario. This suggests that barriers to optimal practice are not simply knowledge-based but are influenced by broader behavioural and systemic factors. Importantly, the present findings align with recent literature highlighting the complexity of real-world sepsis recognition, where individual knowledge, professional confidence, operational protocols, and organisational culture intersect [29, 48].

## Knowledge

Notably, this survey was purposefully designed to assess the practical application of sepsis knowledge in realistic clinical scenarios, rather than to test theoretical recall. Although most practitioners recognised sepsis as a time-critical emergency, significant gaps persisted in determining whether sepsis was present in clinically contrasting presentations. In keeping with international trends, scenario-based items revealed inconsistency in determining whether sepsis was present, with respondents performing substantially better in the more overt septic scenario than in the first, clinically ambiguous non-sepsis scenario, reflecting a diagnostic threshold bias [49, 50]. The lack of a validated, context-appropriate pre-hospital screening tool, combined with the inherent variability in sepsis presentations, likely contributes to this challenge.

Although three-quarters of respondents reported receiving prior sepsis training, the depth and recency of training varied across participants. While such training likely improves awareness of sepsis definitions and clinical indicators, it does not necessarily translate into accurate application. While differences were observed in selected scenario-based items across clinical role and experience, overall knowledge scores did not differ significantly between groups, indicating variability at an item level rather than across composite performance.

Participants struggled in three key areas: accurately determining whether sepsis was present in the first scenario-based question (Q21), acknowledging that sepsis may be present in the absence of fever or an obvious source of infection (Q26), and correctly identifying the current classification of sepsis, with many still referencing the outdated *severe sepsis* category (Q30) (Table 3). This echoes longstanding concerns that conventional training may prioritise rote knowledge over nuanced, scenario-driven decision-making [51].

Respondents reported relying heavily on clinical judgement and experience, with structured screening tools such as qSOFA applied inconsistently and typically in a confirmatory rather than case-finding capacity (See Appendix B, Figure 1). This reliance on gestalt and tacit knowledge is not unique to Qatar’s setting, but may introduce subjectivity and variability in recognition thresholds [48, 52].

Nevertheless, the efficacy of these tools is limited, as demonstrated by other research findings [6]. For example, qSOFA has shown poor sensitivity for pre-hospital identification of severe sepsis, with one study reporting only 16.3% sensitivity, indicating a high risk of missed cases, despite 97.3% specificity [6]. Another study has revealed that PRESS had only 11% sensitivity with a positive predictive value of 56%, while PRESEP achieved 49% sensitivity with a positive predictive value of 36%, underscoring the performance limitations of these screening tools [53]. These local findings mirror wider prehospital evidence: recent reviews and large UK ambulance cohort data similarly report highly variable sensitivity and specificity across tools, and show that no single screening strategy combining early warning scores with paramedic impression can achieve both high sensitivity and acceptable positive predictive value for sepsis. This reinforces the conclusion that screening tools should support, rather than replace, clinical judgement in pre-hospital care [23].

Variation in applied knowledge was more strongly associated with role-specific exposure and training pathways for individual items. This supports a targeted educational approach that focuses on real-world scenarios and critical items, rather than generic knowledge reinforcement.

## Attitude

Broadly, participants demonstrated a positive attitude towards sepsis recognition, expressing strong beliefs in the critical role of PHEMC in patient outcomes and their ability to recognise sepsis in pre-hospital care. Confidence in diagnostic ability was widely reported, with CCPs exhibiting stronger attitudes, likely reflecting greater experience, broader clinical scope, and higher professional autonomy. This pattern aligns with previous studies, which have demonstrated a close link between the perceived importance of PHEMC and self-reported confidence [29, 31]. This level of confidence might be further explained by the availability of well-established clinical practice guidelines in HMCAS, which, in other settings, has been shown to be a helpful tool that enhances PHEMC attitudes toward clinical decision-making [54]. The high explanatory power of the multiple linear regression model (R^2^ = 0.657) further suggests that these attitudinal, training-related, and experiential factors were strongly associated with self-reported diagnostic confidence in this cohort.

However, this confidence did not consistently translate into accurate early-stage sepsis recognition, revealing a clear confidence–competence gap. Seymour et al. [31] similarly observed that EMS personnel often reported high awareness despite uneven diagnostic performance. Recent literature suggests that effective clinical decision-making requires a close alignment between self-confidence and actual ability [55]. Misalignment, whether due to overconfidence or underconfidence, can impair judgment and limit learning opportunities, especially in high-autonomy, low-feedback environments such as pre-hospital care.

Another relevant factor is the influence of hierarchy and organisational safety culture on the expression of confidence. While CCPs demonstrated higher confidence and more proactive attitudes, many practitioners, especially APs and CCAs, reported uncertainty about the adequacy of their sepsis training and greater diagnostic hesitation.

Those who had previously missed a sepsis diagnosis often reported lower confidence, suggesting that reflective learning, caution after setbacks, and concern about reputational scrutiny may temper clinical self-assurance (Table 5), as also identified by a recent study in Hamad Medical Corporation in Qatar exploring factors influencing patient safety culture [56]. Literature on psychological safety supports this interpretation, highlighting that expressions of confidence are often context-dependent and shaped by organisational culture and professional hierarchies [55]. Ghazal et al. [29] found that engagement in pre-hospital sepsis care was associated with higher confidence and more positive attitudes, supporting the influence of both psychological and organisational factors on clinical behaviour.

Importantly, participants expressed their enthusiasm for system-level improvements, particularly pre-notification protocols and rapid escalation, reflecting the value placed on early intervention (Fig. 1). This mirrors findings by Polito et al. [30], who reported widespread support among EMS personnel for pre-hospital alerts and prioritisation of sepsis alongside other high-acuity emergencies.

Nonetheless, more cautious attitudes toward pre-hospital antibiotic administration were expressed (Fig. 1). This divergence suggests that while practitioners are confident in recognising sepsis, they may be more cautious when initiating definitive treatment. Polito et al. [30] reported similar concerns, with EMS providers citing scope of practice, medicolegal risk, and interprofessional dynamics as barriers to initiating treatment. These insights indicate that readiness to intervene is shaped as much by institutional clarity, role definition, and system-level permissions as by individual confidence.

Overall, the attitudes of PHEMC practitioners reflect a motivated and reflective workforce. However, the translation of clinical suspicion into consistent action is influenced by more than knowledge alone; factors such as confidence calibration, professional hierarchy, and organisational culture play a significant role. Addressing these influences will require more than conventional sepsis training. As suggested by Seymour et al. [31] and Park et al. [8], interventions that combine targeted education, structured reflection, clear protocols, and supportive feedback mechanisms may help bridge the gap between perception and practice. Furthermore, Gottlieb et al. [55] highlight that fostering psychologically safe environments and encouraging ongoing calibration of confidence can support accurate self-assessment and promote more consistent clinical behaviour in pre-hospital sepsis care.

## Practice

The practice behaviours of PHEMC practitioners revealed a tendency to rely more heavily on clinical judgement and patient presentation than on routine use of screening tools. Tools such as qSOFA were recognised but typically applied selectively, often only after sepsis was already suspected. This reactive, confirmatory approach limits the effectiveness of screening tools in detecting early or ambiguous cases, ultimately narrowing the window for timely intervention [21].

International literature has reported similar findings. Hilditch [57] found that UK paramedics frequently applied screening tools only after clear signs of deterioration, relying instead on clinical gestalt. Similarly, Ludick et al. [21] reported inconsistent tool use despite their known potential to improve recognition. These findings point not to a lack of access, but to the absence of consistent, structured application.

Differences in practice behaviours were observed by clinical scope and training, with higher-level practitioners more likely to engage in structured recognition and escalation behaviours. Practitioner experience was also commonly identified by respondents as an influence on sepsis recognition, although years of experience did not show consistent inferential differences across practice outcomes.

In this study, sepsis was not consistently prioritised as a leading differential in undifferentiated patients. While most practitioners acknowledged its importance, many hesitated to consider it without overt physiological compromise, a bias also evident in the knowledge domain (Table 3). Recognition was substantially better in the more overt septic scenario than in the first scenario, suggesting that diagnostic suspicion is often anchored in clearer signs of deterioration. This pattern indicates difficulty not only in recognising sepsis when it is clinically overt, but also in accurately determining when sepsis is absent in more ambiguous presentations. Similar patterns have been reported in other EMS systems, where prehospital suspicion or documentation of sepsis is recorded in only a minority of patients later diagnosed in hospital, despite the substantial sepsis burden within EMS caseloads [19, 25].

This tendency suggests that current screening practices are often driven by diagnostic certainty rather than proactive clinical vigilance, thereby undermining the intended role of these tools. Ultimately, a behavioural pattern emerges in which PHEMC practitioners respond to sepsis once it becomes clinically overt, rather than actively screening for its early signs.

Pre-notification behaviours were similarly variable and appeared strongly influenced by institutional knowledge. Practitioners familiar with sepsis alert protocols reported more consistent communication with hospitals, while others were unsure whether such systems existed or how to activate them (Table 6). This inconsistency appears rooted more in operational ambiguity than in individual clinical hesitancy. Comparable observations in other EMS systems have attributed missed escalation opportunities to poor protocol visibility or accessibility [58].

Findings from the results further indicated that recognition was predominantly guided by clinical judgement and patient presentation rather than structured screening criteria, reinforcing the dominance of intuitive reasoning in pre-hospital sepsis care.

This divergence between generally positive attitudes and self-reported knowledge, and cautious or inconsistent practices, illustrates a central tension: that awareness and intent alone are insufficient without systems that translate them into routine behaviour. Despite recognising sepsis severity and expressing a willingness to improve care, many practitioners defaulted to variable, experience-based approaches. This underscores a behavioural inertia that is unlikely to be resolved through education alone. Instead, sustained change will require protocol reinforcement, system clarity, and feedback mechanisms that promote proactive action.

## System-level drivers of practice behaviour

Perhaps the most significant insight from the practices domain is that system-level factors appear to shape sepsis recognition more than individual clinical capability. In Qatar, institutional pathways for conditions like myocardial infarction and stroke are well established, underpinned by embedded protocols and automatic pre-alert systems. In contrast, escalation pathways for suspected sepsis remain underdeveloped. The lack of standardised screening tools or consistent pre-notification processes may limit timely action—even among well-trained providers.

By reframing sepsis recognition as a system challenge rather than a deficit at the individual clinician level, this study highlights the need for institutional scaffolding: formalised screening expectations, reliable alert mechanisms, and a workplace culture that normalises early clinical suspicion. Bridging the gap between knowledge, attitude, and practices will require coordinated efforts across policy, training, and operational support.

## Limitations

The study is limited by its single-centre design; therefore, the findings may not be generalisable to regions with different operational, cultural, or resource contexts. The sample was also predominantly male (90%), which largely reflects the gender distribution of the HMCAS PHEMC workforce but limits exploration of gender-related differences in sepsis recognition.

The achieved sample (*n* = 221) was below the pre-specified minimum (*n* = 288) based on a precision calculation, which may reduce the precision of estimates and the ability to detect smaller subgroup effects. In addition, the response rate introduces the potential for non-response bias, as the characteristics of non-responders could not be evaluated due to the anonymous survey design.

The reliance on self-reported data introduces potential recall and social desirability bias. Although scenario-based items aimed to capture applied knowledge, they may still reflect perceived rather than actual behaviour. The cross-sectional design limits causal inference.

While exploratory regressions were used to identify associations, multiple comparisons were conducted without formal adjustment for multiplicity, increasing the risk of Type I error; therefore, these findings should be interpreted as exploratory and hypothesis-generating rather than confirmatory.

In addition, screening tools were assessed for usage patterns, but not for diagnostic accuracy. Similarly, although the questionnaire demonstrated acceptable content validity and internal consistency, it has not yet undergone full psychometric validation across independent EMS populations (e.g. factor analysis and external validation), and these measurement properties should be confirmed in future work.

Furthermore, although knowledge scores were categorised into predefined levels, these thresholds should be interpreted cautiously, as performance varied across individual items, particularly in scenario-based assessments of whether sepsis was present across contrasting clinical presentations.

Future research should address these limitations through multi-centre designs, direct observation of clinical practice, or studies that incorporate real-time decision-support interventions.

## Conclusion

This study suggests that knowledge deficits alone may not fully explain the challenges of pre-hospital sepsis recognition. Despite Qatar’s highly trained, multidisciplinary EMS workforce, scenario-based determination of whether sepsis was present remained inconsistent, with decision-making appearing to be influenced by overt physiological deterioration rather than proactive screening. Confidence levels did not consistently align with diagnostic accuracy, although this should be interpreted cautiously given the achieved sample of 221 against a target of 288 participants and the 21.6% response rate. Although screening tools were known, they were rarely integrated into routine workflows. These trends reflect a broader disconnect between knowledge, attitudes, and frontline behaviour. Importantly, this misalignment appears to be shaped not only by individual readiness, but also by system-level design. Structural enablers present for other emergencies (e.g. STEMI, stroke) are not mirrored for sepsis, which may limit institutional signalling and undermine consistent escalation. These findings suggest that future improvement depends not only on education and training, but also on aligning operational culture, clinical pathways, and diagnostic expectations.

The challenges observed are consistent with those reported internationally in similarly structured EMS systems. Addressing the gap between sepsis knowledge and timely pre-hospital action will require coordinated changes in protocol development, system design, and frontline practice, rather than further training alone. Collectively, these findings support reframing pre-hospital sepsis care as a systems challenge rather than an individual knowledge deficit. Future research should explore the lived realities and organisational determinants that shape frontline decision-making in order to develop practical, context-appropriate strategies that improve recognition and patient outcomes.

## Electronic supplementary material

Below is the link to the electronic supplementary material.

Supplementary material 1

## Data Availability

The datasets generated and analysed during the current study are not publicly available due to participant confidentiality and institutional restrictions. De-identified data may be made available from the corresponding author on reasonable request and with approval from Hamad Medical Corporation and the University of Edinburgh.
