# Supplementary material for: Knowledge, attitudes, and practices of pre-hospital emergency medical care practitioners regarding sepsis recognition: a cross-sectional study in Qatar
Source: BMC Emerg Med. 2026 May 16;26:192. doi: 10.1186/s12873-026-01607-7 (PMC13352874; doi:10.1186/s12873-026-01607-7)
Supplement: Supplementary file 1 — Supplementary material 1 [file 12873_2026_1607_MOESM1_ESM.docx]

**Appendix A: Survey Questionnaire**

The following appendix reproduces the survey questionnaire used for data collection. Survey-platform administrative pages, consent acknowledgement screens, post-survey invitation pages, and submission instructions have been omitted for brevity. Item wording and response options are presented as administered.

**Section 1. Demographic Information**

1. Age Category

- 20–30
- 31–40
- 41–50
- Over 50

2. Gender

- Male
- Female
- Choose not to disclose

3. Are you currently practising as a pre-hospital emergency medical care (PHEMC) practitioner in Qatar?

- Yes
- No

3a. How would you qualify your primary role/function at Hamad Medical Corporation Ambulance Service (HMCAS)?

- Operations
- Administrative/Support
- Training

4. Are you registered with the medical board in Qatar (Qatar Council for Healthcare Practitioners [QCHP])?

- Yes
- No

5. What is your current scope/level of practice?

- Ambulance Paramedic (AP)/Critical Care Assistant (CCA)
- Critical Care Paramedic (CCP)

6. How many years of experience in total do you have as a licenced PHEMC practitioner?

- 2 or less years
- 3–5 years
- 6–10 years
- 11 or more years

6a. How many of these years of experience as a licensed PHEMC practitioner were gained while working in Qatar?

- 2 or less years
- 3–5 years
- 6–10 years
- 11 or more years

7. What is the highest medical qualification you hold?

- No formal qualification
- Certificate
- Diploma
- Associate degree
- Bachelor's degree
- Master's degree
- Medical doctor
- Doctor of Philosophy (PhD)
- Other

8. In addition to your current licensure as a PHEMC practitioner, have you ever been or are currently licensed to practice in any other medical profession?

- Yes
- No

If 'Yes', please specify: __________

8a. Do you have any speciality in which you have practised within this other profession?

- Yes
- No

If 'Yes', please specify: __________

9. Within your training or education as a healthcare professional, have you received training to recognise patients with sepsis?

- Yes
- No
- Unsure

9a. What is the level of training or education you have received on sepsis recognition?

- Basic (1/2 day course)
- Intermediate (2-day course)
- In-depth (full university module)
- Unsure

9b. When last did you receive training on sepsis recognition?

- Within the last 6 months
- Within the last year
- Within the last 2 years
- More than 2 years ago
- Unsure

**Section 2. Knowledge–Attitude–Practices**

*2.1 Attitude-Based Assessment*

Please rate each statement based on your level of agreement using the following scale:

- Strongly disagree
- Disagree
- Unsure
- Agree
- Strongly agree

2.1.1. As a PHEMC practitioner, you are confident in your ability to make a sepsis diagnosis promptly and accurately.

2.1.2. You have received up-to-date training on sepsis recognition, consistent with recognized standards or guidelines, which makes you confident in your recognition and management of patients with sepsis.

2.1.3. Within the chain of care for sepsis patients, pre-hospital emergency medicine is a vital link impacting patient outcomes.

2.1.4. The accurate diagnosis and management of sepsis patients by you, as a PHEMC practitioner in the pre-hospital setting, significantly impacts sepsis patient outcomes.

2.1.5. You believe that communicating your suspicion of sepsis to the emergency department (ED) staff will be taken seriously and can positively influence the quality and timeliness of patient care by the ED staff.

2.1.6. Because it is not a diagnosis commonly thought of, it is possible that you could have missed a patient having sepsis, even in the presence of clear signs and symptoms.

2.1.7. You sometimes feel reluctant to make a diagnosis of sepsis or communicate your suspicion of sepsis in a patient due to the fear of potentially being wrong and, therefore, facing judgment.

2.1.8. Pre-notification of a sepsis patient to the receiving hospital can improve patient care and outcome.

2.1.9. The administration of antibiotics for the treatment of sepsis should be implemented in pre-hospital emergency medicine.

2.1.10. As a healthcare practitioner, when you suspect sepsis in a patient, it is necessary for you to have the same sense of urgency and considerations for diagnosis, treatment, and transportation as you would have for a patient with an ST-elevation myocardial infarction (STEMI) or stroke.

2.1.11. As a PHEMC practitioner, you feel you require more knowledge and training regarding sepsis recognition.

2.1.12. You consider sepsis (not specifically septic shock) a life-threatening medical emergency.

*2.2 Practice-Based Assessment*

2.2.1. Are you currently using any screening tool to aid your diagnosis of sepsis in the pre-hospital clinical setting?

- Yes
- No

2.2.1a. Do you apply the screening tool to every patient or only to patients you suspect might have sepsis?

- All patients
- Suspected sepsis patients only

2.2.2. In patients who are critically ill with an undifferentiated diagnosis, sepsis is one of your top three differential diagnoses.

- Strongly disagree
- Disagree
- Unsure
- Agree
- Strongly agree

2.2.3. A clear guideline indicating which patients should be screened for sepsis should be made available to PHEMC practitioners.

- Yes
- No
- Makes no difference
- Unsure

2.2.4. Does the current system you work in have a formal policy or procedure for pre-notifying the receiving hospital about a suspected sepsis patient?

- Yes
- No
- I don't know; I'm willing to learn.

2.2.5. As a PHEMC practitioner, do you pre-notify the receiving healthcare facility of a patient with suspected sepsis?

- Never
- Rarely
- Sometimes
- Often
- Always

2.2.6. Diagnosing sepsis is difficult as the clinical signs and symptoms are often nonspecific.

- Strongly disagree
- Disagree
- Unsure
- Agree
- Strongly agree

2.2.7. What factors do you think primarily heighten your index of suspicion for sepsis? *Please select all that apply.

- Patient's clinical presentation
- Experience
- Clinical judgement
- Sepsis screening tool
- Source of infection
- Patient's response to treatment
- Other

If 'Other', please specify: __________

2.2.8. From your experience, what real factors may influence sepsis recognition in the pre-hospital clinical setting by PHEMC practitioners? *Please select all that apply.

- Training and education
- Practitioner Attitude
- Practitioner experience
- Contact time between the patient and practitioner
- Practitioner awareness
- Availability of diagnostic tools
- Complexity of sepsis
- None
- Other

If 'Other', please specify: __________

2.2.9. Would you say you may tend to diagnose septic shock more frequently than cases of only sepsis, as the symptoms of septic shock are typically more evident and recognizable?

- Yes
- No
- Unsure

*2.3 Knowledge-Based Assessment*

Scenario-based questions

Case Scenario 1

History: You are presented with a 26-year-old female with a history of recurring urinary tract infection (UTI). The patient has been complaining of progressive dysuria, urgency, nausea, vomiting, acute pain to the left lower quadrant (LLQ) and fever for the last two days. The patient describes the pain as sharp and constant, with a pain score of 6/10. The patient has no past medical history and is not currently taking any medication.

On examination, you find the following: On palpation, the abdomen is soft, non-distended, with LLQ tenderness and no guarding. The patient describes the pain as sharp and constant. Nothing makes the pain better or worse.

Vital signs:

Temperature: 38.1°C

Heart rate: 131 bpm

Blood pressure: 96/54 mmHg

Respiratory rate: 20

Oxygen saturation: 95% on room air

HGT: 6.5 mMol

Treatment: The patient responds well to intravenous (IV) Paracetamol and Penthrox for the pain. After 500 ml of IV fluids, the patient's vitals remain unchanged.

2.3.1. Do you think the patient presented to you in Scenario 1 has sepsis?

- Yes
- No
- I don't know; I'm willing to learn

2.3.2. Please select your scope of practice to present the following question.

- AP/CCA
- CCP

a. Do you feel it is necessary to call CCP for backup for this patient?

- Yes
- No
- Unsure

b. Do you think it is necessary to administer Noradrenaline to this patient?

- Yes
- No

Case Scenario 2

History: On arrival, you find a 48-year-old female patient presenting with an altered mental status and general body weakness. The history reveals that the patient was at the hospital for an elective, complete hysterectomy. The patient is five days post-surgery and was doing well but is currently confused. The patient lives alone, and the family reports that the patient smokes approximately ten cigarettes daily. The patient has a past medical history of hypertension, hypothyroidism and angina pectoris, all of which have been stabilised on medications.

On examination, you find the following: The patient has a GCS of 14/15 (E4V4M6) and is short of breath. On auscultation, the patient has coarse crackles in her left mid-lower chest. Furthermore, the surgical wound seems to be healing well, and her abdomen is soft and non-tender.

Vital signs:

Temperature: 38.9

Heart rate: 128 beats/min

Respiratory rate: 34 breaths/min

Oxygen saturation: 89% on room air

Blood pressure: 111/58

2.3.3. Do you think the patient presented to you in Scenario 2 has sepsis?

- Yes
- No
- I don't know; I'm willing to learn

2.3.4. When evaluating whether the patient in Scenario 1 and 2 had sepsis, what did you primarily base your decision on? *Please select the most applicable option.

- specific sepsis criteria (e.g., SIRS, qSOFA, QEWS, NEWS)
- Use of a sepsis screening tool
- Training and education in sepsis recognition
- personal experience or judgement
- Other factors
- Unsure

Which sepsis criteria? __________

Which sepsis screening tool? __________

If you have selected 'Other Factors', please specify: __________

2.3.5. Which pre-hospital sepsis screening tools have you heard of, never heard of, are you familiar with, have you used, or are you regularly using?

- For each listed tool, select one: Never heard of it / Heard of it / Familiar with / Used it / Regularly using

BAS 90-30-90

PRESEP – Pre-hospital Early Sepsis Detection

PRESS – Pre-hospital severe sepsis score

PreSAT – Pre-hospital Sepsis Assessment Tool

qSOFA – Sequential Organ Failure Assessment

Other

If 'Other', please specify any screening tools not listed: Other 1 __________ / Other 2 __________ / Other 3 __________

For each 'Other' screening tool provided, select one: Never heard of it / Heard of it / Familiar with / Used it / Regularly using

2.3.6. Would you still consider the possibility of sepsis in any of these two patients if the patients did not present with fever and an obvious source/history of infection?

- Yes
- No
- Unsure

2.3.7. Based on the two scenarios presented, do you think early identification of sepsis would change the treatment you provide to the patients?

- Yes
- No
- Unsure

2.3.8. Assuming pre-hospital antibiotic treatment is within your scope of practice and you suspected sepsis in either Patient A, Patient B, or both from Scenario 1 and 2, what would your course of action be?

- Administer antibiotics immediately based on clinical suspicion.
- Initiate other supportive treatments but hold off on antibiotics until a formal diagnosis is established with laboratory tests at hospital.
- Unsure

2.3.9. Considering the scenarios of the two patients you treated, do you think obtaining blood cultures and serum lactate levels in the pre-hospital setting would be beneficial in these patients' chain of care?

- Yes
- No
- I don't know; I'm willing to learn

2.3.10. If a patient presents with sepsis, it is common practice today to categorise them into the following different levels, namely “sepsis”, “severe sepsis”, and “septic shock”.

- Yes
- No
- I don't know; I'm willing to learn

2.3.11. Do you believe that if a patient with sepsis is not treated, they will almost certainly develop septic shock?

- Yes
- No
- I don't know; I'm willing to learn

2.3.12. Generally, which treatments are essential for managing patients with sepsis?

- a) Antibiotics, fluid resucitation, tight glucose control, source control, and vasopressors (inotropic support)
- b) Fluid resucitation, intubation, tight glucose control, and ventilation, and vasopressors (inotropic support)
- c) Antibiotics, fluid resucitation, Diuretics, and Vasopressors (inotropic support)
- d) Fluid resuscitation, vasopressors (inotropic support), and corticosteroids
- e) Antibiotics, fluid resucitation, vasopressors (inotropic support), and source control
- I don't know; I'm willing to learn

2.3.13. I believe sepsis has a higher global mortality rate than the following conditions: *Please select all that apply.

- Lung Cancer
- Stroke
- Major Trauma
- Myocardial Infarction
- COPD (Chronic obstructive pulmonary disease)
- None of the above
- I don't know; I'm willing to learn

2.3.14. When do you think sepsis in patients is often first suspected/identified?

- Pre-hospital phase of care
- During the initial evaluation in the emergency department (ED)
- After additional laboratory tests in the ED
- Intensive care unit
- Unsure

2.3.15. In your opinion, would the implementation of dedicated sepsis pre-notification pathways be beneficial to this patient group?

- Yes
- No
- Unsure

2.3.16. Do you agree with the statement: The management of sepsis patients is time-critical, irrespective of their specific condition?

- Yes
- No
- I don't know; I'm willing to learn

14. The below question is presented in a grid format and consists of two parts in total to be answered.

| 1. Part One: Choosing Signs and Symptoms   Below, you'll find 15 options.  In 'Part One: Please Select', pick the top six signs and symptoms that would make you suspect a patient might have sepsis early on.  Please select exactly 6 answer(s).   - Source of infection - Cold extremities - Chest pain - Malaise - Abdominal pain - Hypothermia - Tachypnea - Fever - Tachycardia - Hypotension - Dizziness - Low end-tidal carbon dioxide (ETCO2) - Hyperglycemia in the absence of diabetes - Altered mental status - Low oxygen saturation - Unsure   Based on the signs and symptoms you identified in Part One as early indicators of sepsis, what factor do you believe mostly influenced your ability to recognize these?   - Specific sepsis criteria/screening tool - Educational Training - Experience - Unsure |
| --- |
| 1. Part Two: Rank Your Chosen Signs and Symptoms   For each sign or symptom you picked in Part One, please rank its likelihood of pointing to sepsis. You can give the same rank to more than one sign or symptom.   - Ranking scale: 1 = Somewhat Likely; 2 = Moderately Likely; 3 = Likely; 4 = Very Likely; 5 = Extremely Likely.   Please rank each selected sign or symptom.  38. Are there any other signs or symptoms not listed in Part One that you'd like to include?   - Yes - No   If yes, list up to four additional signs or symptoms and rank each using the same 1–5 scale. |

**Appendix B: Tables and Figures**

**Tables**

**Table 1***Factors heightening suspicion for sepsis among study participants*

| Factor | Number of Responses (*N*) | Percent of Responses (%) | Percent of Participants (%) |
| --- | --- | --- | --- |
| Patient’s clinical presentation | 207 | 22.2 | 93.7 |
| Experience | 156 | 16.7 | 70.6 |
| Clinical judgement | 160 | 17.1 | 72.4 |
| Sepsis screening tool | 149 | 16.0 | 67.4 |
| Source of infection | 153 | 16.4 | 69.2 |
| Patient’s response to treatment | 98 | 10.5 | 44.3 |
| Other | 11 | 1.2 | 5.0 |
| Total Responses | 934 | 100.0 | – |

**Figure 1***Participating Pre-hospital Emergency Medical Care practitioners reported use of their key diagnostic indicators (n=221)*

**Figure 2***Awareness and Usage of Pre-Hospital Sepsis Screening Tools by the Study Participants*

*Note*: Percentage survey responses of PHEMC practitioners in Qatar.

**Figure 3***Distribution of knowledge level categories (Poor, Adequate, Excellent) by scope of practice (CCP vs AP/CCA)*

*Note*. While CCPs had higher average knowledge scores, no statistically significant difference in overall knowledge level distribution was observed between the two groups.
